# IL-21-mediated non-canonical pathway for IL-1β production in conventional dendritic cells

**DOI:** 10.1038/ncomms8988

**Published:** 2015-08-13

**Authors:** Chi-Keung Wan, Peng Li, Rosanne Spolski, Jangsuk Oh, Allison B. Andraski, Ning Du, Zu-Xi Yu, Christopher P. Dillon, Douglas R. Green, Warren J. Leonard

**Affiliations:** 1 Laboratory of Molecular Immunology and the Immunology Center, National Heart, Lung and Blood Institute, National Institutes of Health, 10 Center Drive, Building 10, Room 7B05, Bethesda, Maryland 20892-1674 USA; 2 Pathology Core, National Heart, Lung and Blood Institute, National Institutes of Health, 10 Center Drive, Building 10, Room 6D19, Bethesda, Maryland, 20892-1674 USA; 3 Department of Immunology, St Jude Children’s Research Hospital, 262 Danny Thomas Place, Memphis, Tennessee, 38105 USA

## Abstract

The canonical pathway for IL-1β production requires TLR-mediated NF-κB-dependent *Il1b* gene induction, followed by caspase-containing inflammasome-mediated processing of pro-IL-1β. Here we show that IL-21 unexpectedly induces IL-1β production in conventional dendritic cells (cDCs) via a STAT3-dependent but NF-κB-independent pathway. IL-21 does not induce *Il1b* expression in CD4^+^ T cells, with differential histone marks present in these cells versus cDCs. IL-21-induced IL-1β processing in cDCs does not require caspase-1 or caspase-8 but depends on IL-21-mediated death and activation of serine protease(s). Moreover, STAT3-dependent IL-1β expression in cDCs at least partially explains the IL-21-mediated pathologic response occurring during infection with pneumonia virus of mice. These results demonstrate lineage-restricted IL-21-induced IL-1β via a non-canonical pathway and provide evidence for its importance *in vivo*.

## Abstract (web summary)

The proinflammatory cytokine interleukin-1ß (IL-1ß) plays an important role in host defence against pathogens. Here the authors report a non-canonical pathway for IL-1 ß production in conventional dendritic cells that is induced by IL-21 via STAT3-dependent mechanism.

## Graphical abstract

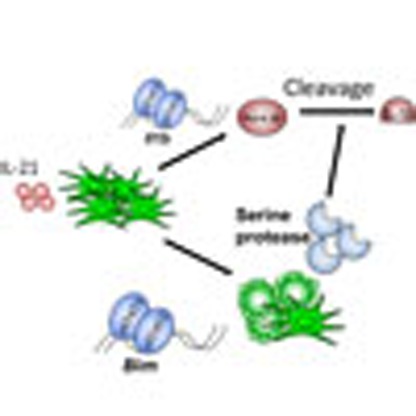

Interleukin-1β (IL-1β) is a cytokine important for host defence to pathogens^1^,^2^. It triggers inflammatory responses by various mechanisms, including the induction of cyclooxygenase-2 and inducible nitric oxide synthase, which control the release of prostaglandin E2 and nitric oxide and recruitment of inflammatory cells^2^,^3^. IL-1β also promotes differentiation of CD4^+^ T cells into Th17 cells^4^, and the production of IL-17 and IL-22 from these cells further recruits neutrophils to the inflammatory sites and stimulates them to release antimicrobial peptides^1^. Local inflammatory responses during pathogen invasion are often beneficial to the host^5^, but they can also be deleterious if not properly controlled. Moreover, dysregulation of IL-1β expression leads to the development of autoimmune diseases, such as rheumatoid arthritis, type 2 diabetes and gout, as well as autoinflammatory diseases, such as familial Mediterranean fever and cryopyrin-associated periodic syndromes^3^,^6^,^7^.

Production of biologically active IL-1β requires immune cells to receive two distinct signals. First, *Il1b* mRNA expression is induced by the toll-like receptor- or IL-1 receptor (IL-1R)-mediated signalling pathways, which activate transcription factor NF-κB, followed by translating the mRNA to the biologically inactive pro-IL-1β (ref. 8). The cells then activate the caspase-1-containing^8^ or the more recently identified caspase-8-containing^9^ inflammasomes when receiving second signals such as bacterial metabolites, with caspase-mediated cleavage of pro-IL-1β into mature IL-1β (ref. 8). However, signals for triggering IL-1β production under conditions of sterile inflammation remain largely unknown, and the mechanism(s) for releasing IL-1β to the extracellular environment remain unclear.

IL-21 is a pleiotropic type 1 cytokine produced by CD4^+^ T cells and natural killer T cells. It promotes immunoglobulin class switch^10^, augments antibody production^10^ and drives terminal differentiation of B cells^11^. IL-21 is critical for the development of multiple forms of autoimmunity in animal models^12^, and antibodies to IL-21 are being tested in phase 1 clinical trials for rheumatoid arthritis (NCT01208506 and EudraCT-2011-005376-42). Moreover, IL-21 exhibits antitumor activity^13^,^14^, with IL-21 in phase 2 clinical trials for various cancers^12^. Although the roles of IL-21 in T- and B-cell function have been extensively studied, there is limited knowledge on the actions of IL-21 on dendritic cells (DCs). IL-21 has been reported to inhibit the lipopolysaccharide (LPS)-stimulated production of several proinflammatory cytokines, including IL-1β, by bone marrow-derived DCs (BMDCs)^15^, and we recently demonstrated that IL-21 is proapoptotic for conventional DCs (cDCs), at least in part by its induction of BIM^16^. Here we show an unanticipated role of IL-21 in inducing IL-1β expression in cDCs. IL-21-induced expression of *Il1b* mRNA is independent of NF-κB activation but requires STAT3. Moreover, we found that this IL-21-STAT3 pathway for regulating IL-1β expression is DC-specific, which correlates with the presence of cell type-specific enhancer landscapes. Importantly, IL-21-mediated production of mature IL-1β does not require a second signal for the activation of inflammasomes and in fact is independent of canonical caspase-containing inflammasomes, but it depends on IL-21-mediated cell death, which also requires STAT3 (ref. 16). Finally, we demonstrate that this IL-21-STAT3-dependent IL-1β expression can at least in part explain the pathologic immune response mediated by IL-21 during infection with Pneumonia Virus of Mice (PVM), a mouse model for human respiratory syncytial virus.

## Results

### IL-21-mediated *Il1b* gene expression in cDCs requires STAT3

**Figure 1:**
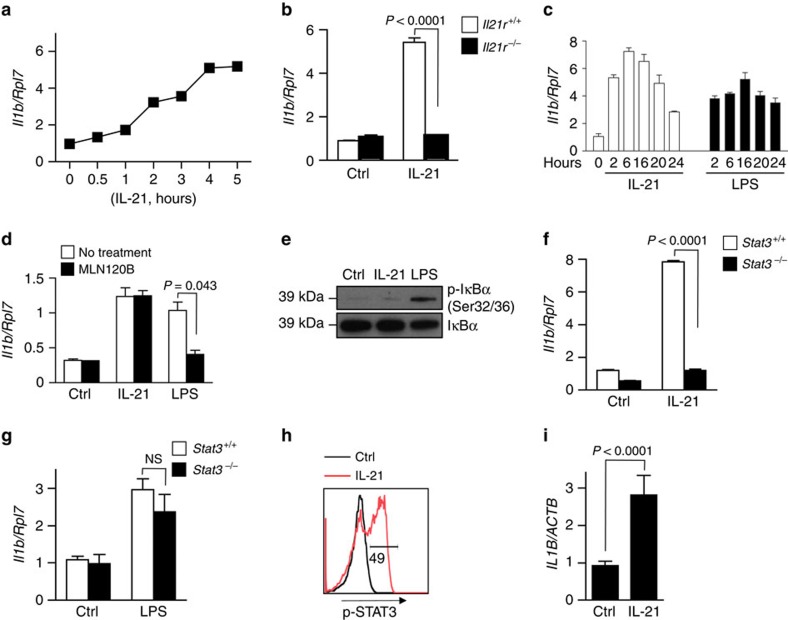
IL-21 induces *Il1b* mRNA expression via STAT3. (**a**) cDCs were rested 1 h, treated with 100 ng ml^−1^ IL-21 at the indicated time points, and *Il1b* mRNA assessed relative to *Rpl7* by reverse transcription–PCR (RT–PCR). Shown is one of two similar experiments. (**b**) *Il21r*^+/+^ and *Il21r*^−/−^ cDCs were treated with IL-21 for 5 h and *Il1b* mRNA assessed. (**c**) cDCs were rested 1 h, treated with 100 ng ml^−1^ IL-21 or LPS at the indicated time points and *Il1b* mRNA assessed relative to *Rpl7* by RT–PCR. Shown are the combined results of two independent experiments. (**d**) cDCs were incubated with 10 μM MLN120B for 1 h, treated with IL-21 or LPS for 4 h and *Il1b* mRNA assessed. (**e**) cDCs were rested 1 h, stimulated with 100 ng ml^−1^ IL-21 or LPS for 30 min, and the expression of phosphorylated and total IκBα was determined. Shown is one of two similar experiments. (**f**,**g**) cDCs from *Stat3*^+/+^ and *Stat3*^−/−^ mice were treated with IL-21 (**f**) or LPS (**g**) as in **b**, and *Il1b* mRNA assessed. In **g**, NS, *P*=0.3. Data in **b** and **d**–**g** are representative of three experiments; error bars are technical duplicates of the representative experiment; shown is expression relative to *Rpl7* assessed by RT–PCR. (**h**) Human peripheral blood DCs were rested 16 h and stimulated with IL-21 for 15 min. pSTAT3 in CD1c^+^ cells was measured by flow cytometry. Shown are data representative of five samples in three experiments. (**i**) Human CD1c^+^ cDCs were rested 16 h, stimulated with IL-21 for 4 h, and *IL1B* mRNA relative to *ACTB* was assessed by RT–PCR. Data are from three experiments (six total samples); error bars are means±s.e.m. Statistical analysis was performed by Student’s *t*-test.

We previously performed microarray analysis to identify genes that are regulated by IL-21 in cDCs^16^. Surprisingly, although IL-21 inhibits the LPS-induced IL-1β expression in BMDCs^15^, we observed a significant induction of *Il1b* mRNA by IL-21 in cDCs. Transcription of *Il1b* mRNA is induced in response to IL-1 itself or microbial products acting via toll-like receptors^2^, but *Il1b* induction by IL-21 was not anticipated. Therefore, we validated this result by reverse transcription–PCR and confirmed that IL-21 indeed rapidly (within 30 min) induced *Il1b* mRNA (Fig. 1a) and that this induction did not occur in *Il21r*^*−/−*^ cells (Fig. 1b), excluding the possibility that it resulted from contaminating endotoxin. We compared the effect of IL-21 with the classical *Il1b*-inducing ligand LPS and found that IL-21 more robustly induced *Il1b* mRNA expression in cDCs than did LPS (Fig. 1c). Because NF-κB is known to be critical for the induction of *Il1b* mRNA expression by LPS^8^, we treated cDCs with an IκB kinase β inhibitor, MLN120B^17^ and this strongly inhibited LPS-induced, but not IL-21-induced, *Il1b* mRNA expression (Fig. 1d), indicating that the effect of IL-21 was independent of NF-κB. Correspondingly, LPS treatment of cDCs induced phosphorylation of IκBα, which promotes its ubiquitination and degradation, and allows nuclear translocation of NF-κB, whereas IL-21 had no effect (Fig. 1e and Supplementary Fig. 1). Because IL-21 can activate STAT3 in cDCs^16^, we next crossed *Stat3-*floxed mice to CD11c-*Cre* transgenic mice to delete *Stat3* in cDCs (herein denoted as *Stat3*^−/−^ mice), and this markedly decreased IL-21-induced (Fig. 1f) but not LPS-induced (Fig. 1g) *Il1b* mRNA expression. Importantly, when we tested human cDCs isolated from peripheral blood, IL-21 also induced tyrosine phosphorylation of STAT3 (Fig. 1h) and augmented *IL1B* mRNA expression (Fig. 1i) in human cDCs. Thus, IL-21 uses a distinctive, STAT3-dependent pathway for the induction of *Il1b* mRNA expression in cDCs.

### Pro-IL-1β induction by IL-21 in cDCs is cell type-specific

**Figure 2:**
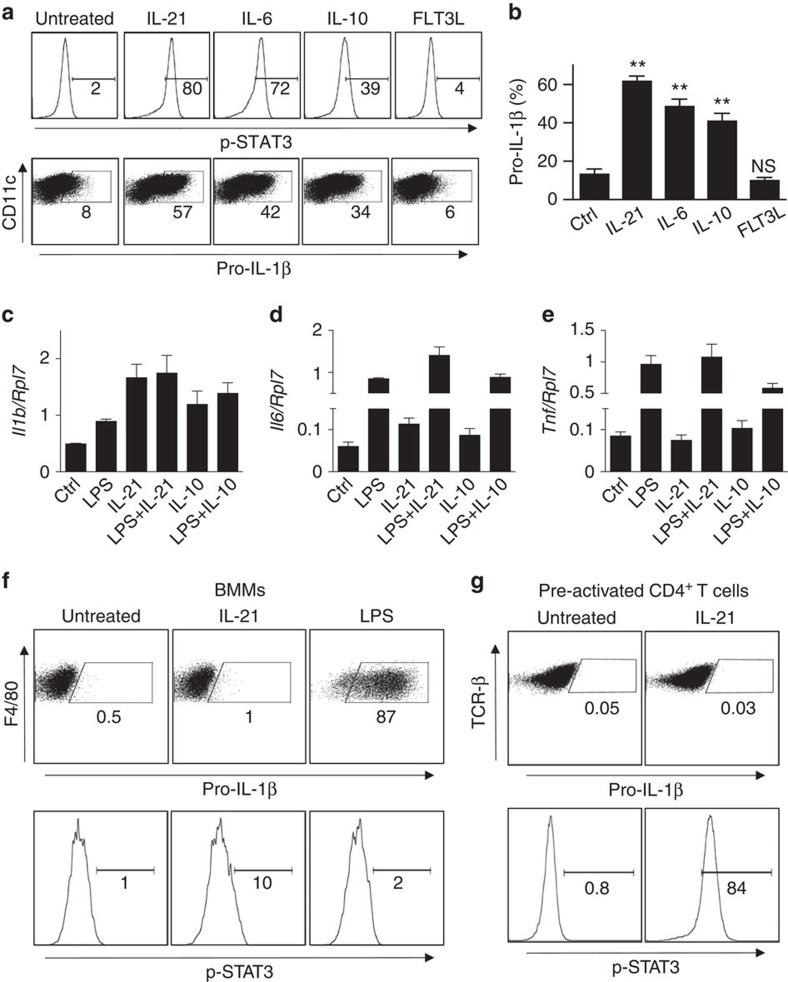
IL-21-induced pro-IL-1β expression is linage-restricted. (**a**) Top panel: cDCs were rested 16 h and stimulated as indicated for 15 min. pSTAT3 was evaluated by flow cytometry. cDCs were gated as CD11c^hi^ cells. Bottom panel: cDCs were rested 1 h, treated with 20 ng ml^−1^ IL-21, IL-6, IL-10 or Flt3L for 4 h, and intracellular pro-IL-1β analysed by flow cytometry. Shown are data representative of three experiments. (**b**) Summary of three experiments from lower panel of **a**. **P values of the untreated sample compared with IL-21, IL-6 and IL-10 treated samples are 0.0002, 0.0017 and 0.0049, respectively; NS, *P*=0.4; error bars are means±s.e.m. (**c**–**e**) cDCs were stimulated with 100 ng ml^−1^ IL-21 or IL-10 for 1 h, then stimulated with 100 ng ml^−1^ LPS for 4 h, and the expression of *Il1b* (**c**), *Il6* (**d**), and *Tnf* (**e**) mRNA were determined. Shown are combined results of 3 independent experiments; error bars are means±s.e.m. (**f**) Top panel: BMMs were rested without M-CSF for 16 h, treated with IL-21 or LPS for 4 h, and intracellular pro-IL-1β assessed by flow cytometry. Bottom panel: BMMs (gated as CD11c^+^F4/80^+^ cells) were rested and treated with IL-21 or LPS for 15 min. pSTAT3 was evaluated by flow cytometry. Data are representative of two experiments (total of 6 individual samples). (**g**) Top panel: CD4^+^ T cells were pre-activated with 5 μg ml^−1^ plate-bound anti-CD3+2 μg ml^−1^ soluble anti-CD28 for 3 days, washed, rested 16 h, treated with IL-21 for 4 h, and intracellular pro-IL-1β assessed by flow cytometry. Bottom panel: Rested CD4^+^ T cells were treated with IL-21 for 15 min. pSTAT3 was evaluated by flow cytometry. Data are representative of three experiments. Statistical analysis was performed by Student’s *t*-test.

To determine if the effect of IL-21 extended to other cytokines that activate STAT3, we stimulated cDCs with IL-6 and IL-10 and confirmed that these cytokines also induced tyrosine phosphorylation of STAT3 in cDCs (Fig. 2a, upper panel) and correspondingly, these cytokines also induced pro-IL-1β production (Fig. 2a, lower panel, and Fig. 2b) in these cells, with the degree of induction approximately correlating with the magnitude of STAT3 activation. In contrast, FLT3 ligand, a cytokine that promotes cDC survival^18^, did not induce phosphorylation of STAT3 nor induce pro-IL-1β (Fig. 2a,b). IL-21 has been shown to suppress LPS-stimulated IL-1β production in BMDCs^15^, and IL-10 is an anti-inflammatory cytokine and suppressor of cytokine production^19^. We therefore pre-treated cDCs with IL-21 or IL-10 and then determined the effect of LPS on *Il1b* mRNA expression. Unlike the situation for BMDCs, IL-21 did not inhibit LPS-induced *Il1b* mRNA expression in cDCs. Corresponding to the effect of IL-10 on pro-IL-1β expression, IL-10 induced *Il1b* mRNA expression but did not suppress the LPS-induced *Il1b* mRNA expression (Fig. 2c). Indeed, neither IL-21 nor IL-10 suppressed LPS-induced *Il6* (Fig. 2d) or *Tnf* (Fig. 2e) mRNA expression, suggesting that the effects of IL-21 and IL-10 on cDCs are different from those on BMDCs.

To further compare the effects of IL-21 and IL-10 on cDCs, including on LPS-treated cells, we performed RNA-Seq analysis. As expected, the number of genes regulated by LPS was greater than those regulated by IL-21 or IL-10 (Supplementary Fig. 2a and Supplementary Data 1a–c). Both IL-21 and IL-10 strongly activate STAT3 in cDCs, and they shared overlapping but also have distinctive gene regulation patterns (Supplementary Fig. 2b,c and Supplementary Data 1d–f). Among 2,278 genes that were regulated by LPS, only 58 were affected by IL-21, and only 15 out of these 58 genes were repressed by IL-21 (Supplementary Fig. 2d and Supplementary Data 2a), indicating that IL-21 did not globally suppress LPS-induced gene expression in cDCs. IL-10 had a stronger effect than IL-21, with 189 out of 2,278 genes regulated by LPS being affected by IL-10, and among these genes, 90 were repressed (Supplementary Fig. 2e and Supplementary Data 2b). We also compared the effects of IL-21 and IL-10 in regulating LPS-mediated gene expression and found that 89 of 105 genes that were differentially expressed by LPS versus LPS+IL-21 stimulation were also regulated by IL-10 (Supplementary Fig. 2f,g and Supplementary Data 2c–e), and overall, there were more IL-10 regulated genes, suggesting that IL-10 had a more potent suppressive effect than IL-21 on LPS-mediated gene regulation. These data indicate that IL-21 and IL-10 have both overlapping and distinctive effects on cDCs.

We also examined the effect of IL-21 on pro-IL-1β expression in bone marrow-derived macrophages (BMMs) and found that IL-21 minimally induced pro-IL-1β (Fig. 2f, upper panel), correlating with weak activation of STAT3 by IL-21 in these cells (Fig. 2f, lower panel), whereas LPS potently induced pro-IL-1β in BMMs (Fig. 2f). Strikingly, IL-21 did not induce pro-IL-1β in pre-activated CD4^+^ T cells (Fig. 2g, upper panel), despite its strongly activating STAT3 (Fig. 2g, lower panel). Thus, STAT3 activation leads to expression of pro-IL-1β in cDCs, but this effect seems to be cell-type specific.

### Divergent STAT3 binding patterns in cDCs versus CD4^+^ T cells

**Figure 3:**
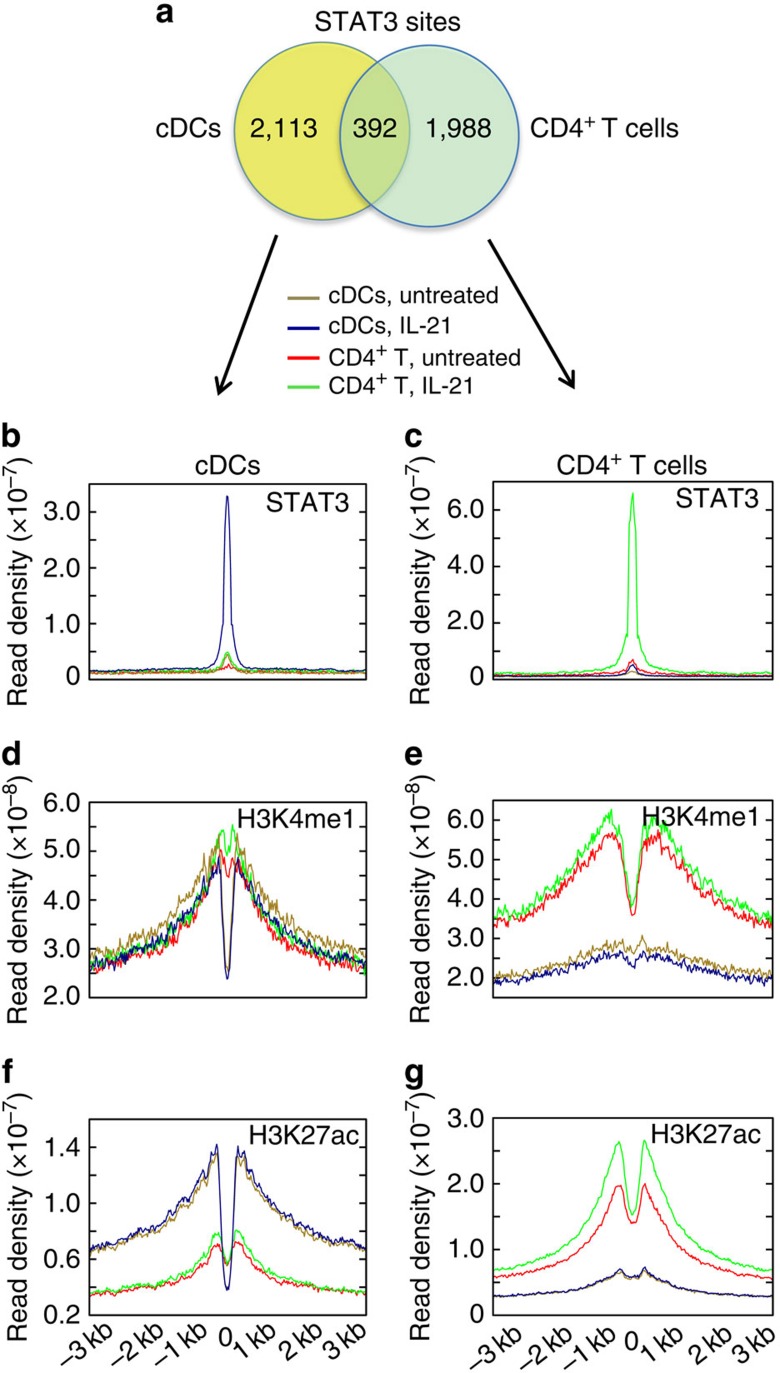
Distinct genome-wide STAT3 binding profile and enhancer landscapes in cDCs and CD4^+^ T cells. (**a**–**g**), cDCs were rested 1 h, treated with IL-21 for 1 h; pre-activated CD4^+^ T cells were washed, rested 16 h, treated with IL-21 for 1 h, and ChIP-Seq performed for STAT3, H3K4me1, and H3K27ac. (**a**) Venn diagram showing overlapping and distinctive STAT3 binding sites in cDCs and CD4^+^ T cells. (**b**–**g**) For IL-21-induced-STAT3 binding sites that differentially exist in cDCs or CD4^+^ T cells, there were cell type-specific binding profiles of STAT3 (**b** versus **c**) and H3K4me1 (**d** versus **e**) and H3K27ac (**f** versus **g**) enhancer marks. Shown are normalized read densities near peak summits for cDC- or CD4^+^ T-cell specific STAT3 binding sites. ‘Dips’ at the plot centres (**d**–**g**) represent open chromatin corresponding to nucleosome depletion. Data are representative of two experiments.

Above, we noted differential effects of IL-21 in cDCs and CD4^+^ T cells, even though IL-21 strongly activates STAT3 in both cell types. We therefore compared the genome-wide STAT3 binding induced by IL-21 in cDCs versus CD4^+^ T cells using ChIP-Seq analysis. Remarkably, STAT3 binding sites in IL-21-stimulated cDCs and CD4^+^ T cells were mostly non-overlapping (Fig. 3a and Supplementary Data 3a,b), with low STAT3 binding in CD4^+^ T cells at sites where IL-21-activated STAT3 binds in cDCs (Fig. 3b, green versus blue lines) and vice versa (Fig. 3c, blue versus green lines). Nevertheless, motif analysis showed that IL-21-activated STAT3 in cDCs and CD4^+^ T cells bound primarily to GAS (gamma-activated site, TTCnnnGAA) motifs (Supplementary Fig. 3). We therefore determined whether the differential STAT3 binding correlated with the presence of active enhancer landscapes. Increased histone H3 lysine-4 mono-methylation (H3K4me1) and H3K27 acetylation (H3K27ac) indicate poised or active enhancer landscapes that promote transcription factor binding^20^, so we compared IL-21-induced STAT3 binding (Fig. 3a–c) with H3K4me1 (Fig. 3d,e) and H3K27ac (Fig. 3f,g) patterns. The ‘dips’ at the center of the histone marks represent open chromatin corresponding to nucleosome depletion that occurs at active promoters and enhancers and promotes transcription factor binding^21^. Sites binding STAT3 in cDCs but not CD4^+^ T cells (Fig. 3b, blue line) had less H3K4me1 (Fig. 3d, blue and gold lines) and H3K27ac (Fig. 3f, blue and gold lines) at the STAT3 peaks but increased H3K27ac adjacent to the STAT3 peaks (Fig. 3f, blue and gold lines), indicating an active enhancer in cDCs but not CD4^+^ T cells. Analogously, sites binding STAT3 in CD4^+^ T cells (Fig. 3c, green line) had less H3K4me1 (Fig. 3e, red and green lines) and H3K27ac (Fig. 3g, red and green lines) at the STAT3 peaks but increased H3K27ac adjacent to the STAT3 peaks (Fig. 3g, red and green lines). These data suggest that STAT3 binding induced by IL-21 in cDCs and CD4^+^ T cells correlates with the presence of active enhancer landscapes.

### Differential epigenetic marks at the *Il1b* and *Il21* loci

**Figure 4:**
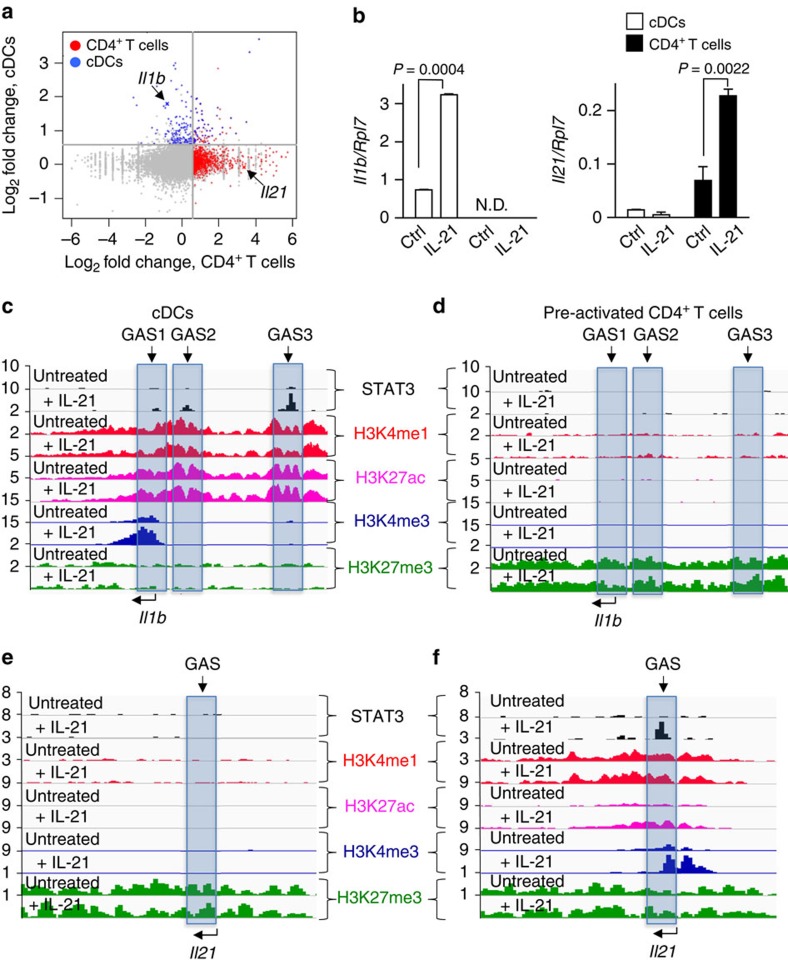
Cell type-specific expression of *Il1b* and *Il21* genes correlates with the differential STAT3 binding and enhancer landscapes. (**a**) Freshly isolated cDCs were rested 1 h, then stimulated with IL-21 for 4 h; CD4^+^ T cells were pre-activated for 3 days, then washed and rested 16 h, and stimulated with IL-21 for 4 h. Shown are genes differentially regulated by IL-21 in cDCs versus pre-activated CD4^+^ T cells. For cDCs, gene expression profiling was performed by microarray analysis, where cDCs were pooled from three independent experiments, as described in ref. 16. For pre-activated CD4^+^ T cells, gene expression profiling data were generated by RNA-Seq analysis. Shown are data from one of two similar experiments. (**b**) *Il1b* and *Il21* expression in cDCs and CD4^+^ T cells not treated or stimulated with IL-21 as in **a**. Data are representative of 3 experiments. Error bars are technical duplicates of the representative experiment. (**c**,**d**) STAT3 binding, H3K4me1, H3K27ac, H3K4me3, and H3K27me3 marks at the *Il1b* locus in cDCs (**c**) and CD4^+^ T cells (**d**). Arrows in **c** indicate STAT3 binding sites at GAS1, GAS2 and GAS3 regions (GAS1: TTAgggGAA (−155 bp), TACcctGAA (−175 bp), TCCctgGAA (−195 bp); GAS2: TTTgggGAA (−2,452 bp), TTCctcCAA (−2,525 bp), TTCttcAAA (−2,549 bp); GAS3: TTGtgtGAA (−9,761 bp)). Arrows in **d** indicate the STAT3 binding sites identified in cDCs, but no STAT3 binding was seen at these sites in CD4^+^ T cells. (**e**,**f**) STAT3 binding, H3K4me1, H3K27ac, H3K4me3 and H3K27me3 marks at the *Il21* gene locus in cDCs (**e**) and CD4^+^ T cells (**f**). Arrow in **f** indicates the STAT3 binding site at the GAS motif in the *Il21* promoter region. Arrow in **e** indicates this same site, but no STAT3 binding was seen at this site in cDCs. Data are representative of two experiments. Statistical analysis was performed by Student’s *t*-test.

The ChIP-Seq results prompted us to determine whether differential STAT3 binding could explain the cell type-specific functions of IL-21 in cDCs and CD4^+^ T cells. Therefore, we compared IL-21-induced gene regulation in these cells, and we found that these STAT3 binding differences correlated with differential IL-21-induced gene expression in cDCs versus CD4^+^ T cells (Fig. 4a) (for example, IL-21 could induce *Il1b* in cDCs but not CD4^+^ T cells, whereas it induced *Il21* in CD4^+^ T cells but not in cDCs (Fig. 4a,b). Interestingly, analysis revealed IL-21-induced STAT3 binding in cDCs at the *Il1b* promoter and 5′ upstream region (Fig. 4c) to GAS-like motifs (Supplementary Table 1), with enriched H3K4me1 and H3K27ac marks at those sites (Fig. 4c), whereas STAT3 did not bind to these sites in CD4^+^ T cells and active enhancer marks were correspondingly absent (Fig. 4d). Conversely, for the *Il21* gene, in cDCs, where the gene is not expressed, neither STAT3 nor H3K4me1/H3K27ac marks were found at the promoter region after IL-21 stimulation (Fig. 4e), but IL-21-activated STAT3 bound to the *Il21* promoter region in CD4^+^ T cells, which express the gene, with enrichment of H3K4me1 and H3K27ac marks (Fig. 4f). Thus, gene expression correlated with the active enhancer landscapes and IL-21-induced STAT3 binding in cDCs versus CD4^+^ T cells. Moreover, we found IL-21-induced H3K4me3 at the *Il1b* promoter (indicating an open chromatin structure^20^) in cDCs where *Il1b* is expressed (Fig. 4c) but not in CD4^+^ T cells where it is not (Fig. 4d); conversely H3K4me3 was strongly present at the *Il21* promoter in CD4^+^ T cells where *Il21* is expressed (Fig. 4f), but not in cDCs where it is not (Fig. 4e). Interestingly, *Il1b* is one of only 24 genes in which we observed much higher H3K4me3 in cDCs than in CD4^+^ T cells (Supplementary Fig. 4). The presence of H3K27me3 repressive marks (indicative of inactive genes^20^) at the *Il1b* locus in CD4^+^ T cells and *Il21* locus in cDCs corresponded to the non-expression of these genes in these cell types. Interestingly, the *Il21* locus in CD4^+^ T cells exhibited both H3K27me3 and H3K4me3 marks (Fig. 4f), indicative of a bivalent gene poised for induction or repression^22^.

### A non-canonical pathway for IL-21-mediated IL-1β production

**Figure 5:**
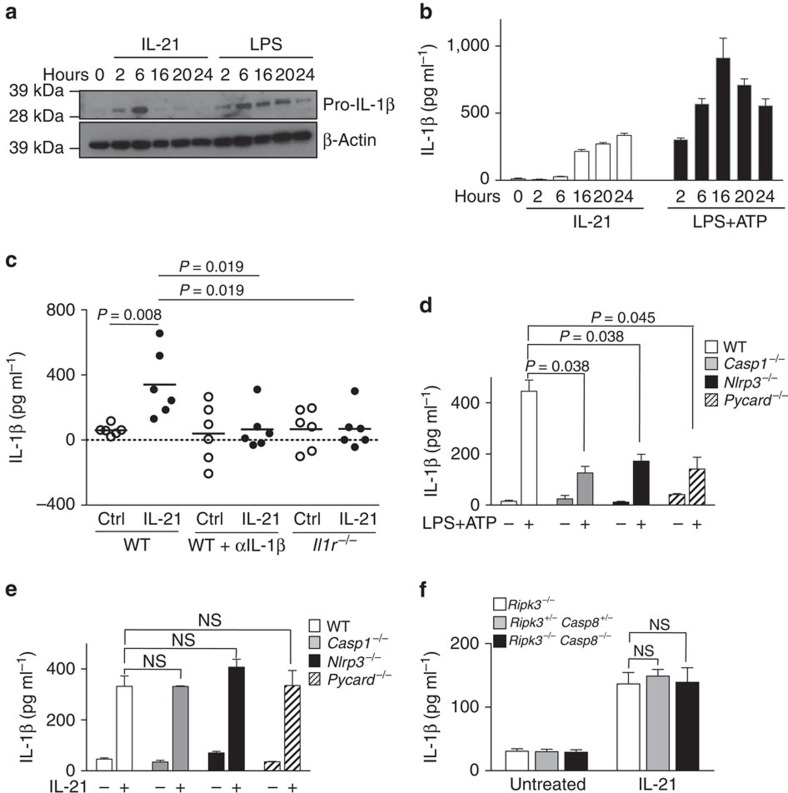
IL-21-induced IL-1β by cDCs is independent of the canonical caspase-containing inflammasomes. (**a**) cDCs were rested 1 h, treated with 100 ng ml^−1^ IL-21 or LPS at the indicated time points, and intracellular pro-IL-1β expression was determined. β-actin was used as control. Shown is one of two similar experiments. (**b**) cDCs were treated as in **a**, with 5 mM ATP added 1 h prior indicated time points in LPS-stimulated samples, and the secretion of IL-1β was determined by enzyme-linked immunosorbent assay (ELISA). Shown are combined results of two independent experiments; error bars are means±s.e.m. (**c**) CD4^+^ T cells from WT or *Il1r*^−/−^ mice were cultured in Th17 cell differentiation conditions for 2 days, then supernatant from a 24 h, IL-21-treated cDC culture was added to the Th17 cells and incubated for 2 days, with or without addition of 10 μg ml^−1^of anti-IL-1β. Expression of IL-2Rα (MFI) was determined by flow cytometry. The amount of biologically active IL-1β was determined using a standard curve constructed by assaying recombinant IL-1β. Shown are the combined results of two independent experiments with total of six samples. (**d**,**e**) WT, *Casp1*^−/−^, *Nlrp3*^−/−^ and *Pycard*^−/−^ cDCs were rested 1 h. In **d**, cDCs were then treated with 100 ng ml^−1^ LPS for 20–24 h with 5 mM ATP added in the final 1 h, and IL-1β assessed. Data are from two experiments; error bars are means±s.e.m. In **e**, cDCs were then treated with IL-21 for 20–24 h and IL-1β protein determined. Data are from five experiments. *P* values of IL-21-treated WT samples as compared with *Casp1*^−/−^, *Nlrp3*^−/−^ and *Pycard*^−/−^ samples are 0.99, 0.22 and 0.96, respectively; error bars are means±s.e.m. (**f**) cDCs from *Ripk3*^−/−^, *Ripk3*^+/−^*Casp8*^+/−^ and *Ripk3*^−/−^*Casp8*^−/−^ mice were treated as in **e**, and IL-1β assessed. Data shown are from three experiments. *P* values of IL-21-treated *Ripk3*^−/−^ sample compared with *Ripk3*^+/−^*Casp8*^+/−^ and *Ripk3*^−/−^*Casp8*^−/−^ samples are 0.57 and 0.93, respectively. In **b** and **d**–**f**, IL-1β production in the culture supernatant was determined by ELISA. Pro-IL-1β induced by IL-21 in the culture supernatant was minimal, based on a pro-IL-1β-specific ELISA. Statistical analysis was performed by Student’s *t*-test.

cDCs develop from precursor cells distinct from those that develop into monocyte-differentiated DCs and macrophages^23^. Whereas IL-1β production has been extensively studied in these latter cells^1^, the molecular machinery for processing pro-IL-1β into mature IL-1β in cDCs has not been reported. We treated cDCs with IL-21 or LPS and determined the expression of pro-IL-1β at different time points. Pro-IL-1β was detected at 2 and 6 h of IL-21 stimulation but then rapidly declined (Fig. 5a and Supplementary Fig. 5). Interestingly, this decline correlated with an increase of secreted IL-1β (from 16- to 24-hour time points, Fig. 5b). The antibodies recognizing secreted IL-1β did not cross-react with pro-IL-1β (Supplementary Fig. 6a,b), and correspondingly, pro-IL-1β was not detected in the cell culture supernatant after IL-21 stimulation (Supplementary Fig. 7), indicating that the protein detected was indeed the mature IL-1β. Importantly, the secreted IL-1β was bioactive and induced IL-2Rα expression in Th17 cells (Fig. 5c). Moreover, this induction was blocked by anti-IL-1β and did not occur in *Il1r*^−/−^ Th17 cells (Fig. 5c), confirming that IL-21 induces production of functional IL-1β.

In macrophages and BMDCs, IL-1β production in response to LPS+ATP involves an inflammasome containing caspase-1, NLRP3 and ASC (apoptosis-associated speck-like protein containing a CARD, encoded by the *Pycard* gene) as critical components. We thus examined IL-1β production in cDCs after stimulation with LPS+ATP, and found that it was greatly diminished in *Casp1*-, *Nlrp3*- or *Pycard*-deficient cDCs (Fig. 5d), indicating that the canonical caspase-1-containing inflammasome is functional in cDCs. Strikingly, however, IL-21-induced production of IL-1β was independent of caspase-1, NLRP3 and ASC (Fig. 5e), indicating a distinctive mechanism. Recently, a caspase-1-independent but caspase-8-dependent non-canonical inflammasome was reported^9^. We therefore, next examined the role of caspase-8 in IL-21-induced IL-1β production using cDCs from *Ripk3* and *Casp8* double-deficient mice, in which RIPK3-dependent necrosis resulting from caspase-8 deficiency is prevented^24^, but we found normal IL-21-induced IL-1β in caspase-8-deficient cDCs (Fig. 5f), indicating that caspase-8 is not essential for IL-21-induced IL-1β production in these cells.

**Figure 6:**
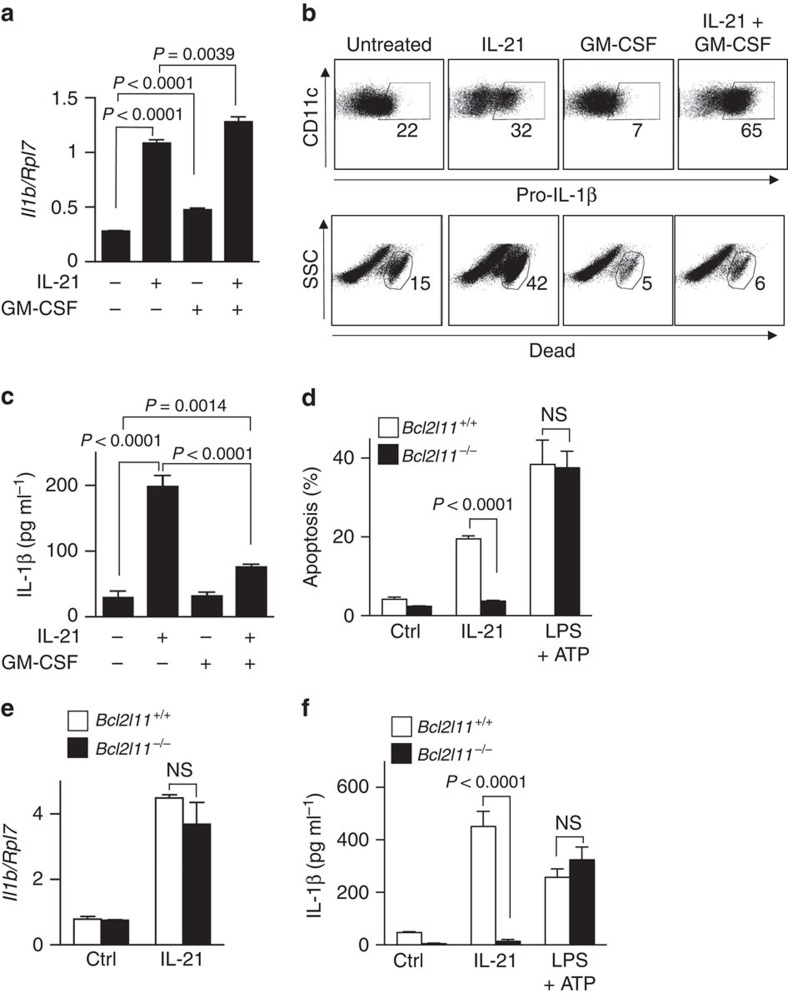
GM-CSF inhibits IL-21-mediated pro-IL-1β processing. (**a**) cDCs were treated with 100 ng ml^−1^ IL-21 and/or 20 ng ml^−1^ GM-CSF for 4 h, and *Il1b* mRNA relative to *Rpl7* assessed by RT–PCR. Data are from three experiments. Error bars are means±s.e.m. (**b**) cDCs were treated as in **a** for 24 h, and intracellular pro-IL-1β expression (top panel) and cell death (bottom panel) analysed by flow cytometry. Data are representative of three experiments. (**c**) cDCs were treated as in **a** for 24 h, and IL-1β protein determined. Data are from three experiments. (**d**) cDCs from *Bcl2l11*^+/+^ and *Bcl2l11*^−/−^ mice were treated with IL-21 for 24 h, or LPS for 24 h with 5 mM ATP added in the final 1 h, and % apoptotic cells (Annexin V^+^ and/or 7-AAD^+^) assessed. NS, *P*=0.84. In **c**,**d**, error bars are means±s.e.m. (**e**) cDCs from *Bcl2l11*^+/+^ and *Bcl2l11*^−/−^ were treated with IL-21 for 4 h, and *Il1b* mRNA relative to *Rpl7* assessed by RT–PCR. Data are representative of three experiments; error bars are technical duplicates of the representative experiment; NS, *P*=0.14. (**f**) As in **d**, but IL-1β protein instead of apoptosis was determined. Data are from three experiments; error bars are means±s.e.m; NS, *P*=0.34. In **c** and **f**, IL-1β levels in the culture supernatant was determined by ELISA. Pro-IL-1β induced by IL-21 in the culture supernatant was minimal, based on a pro-IL-1β-specific ELISA. Statistical analysis was performed by Student’s *t*-test.

Previously, we showed that IL-21-induced apoptosis of cDCs could be rescued by granulocyte-macrophage colony-stimulating factor (GM-CSF)^16^, so we investigated the effect of GM-CSF and whether apoptosis is required for IL-1β production. Interestingly, GM-CSF did not inhibit *Il1b* mRNA expression, and in fact GM-CSF slightly induced its expression, with GM-CSF+IL-21 inducing slightly more *Il1b* mRNA than IL-21 alone (Fig. 6a). However, adding GM-CSF enhanced the accumulation of unprocessed pro-IL-1β (Fig. 6b, upper panel), but GM-CSF significantly decreased IL-21-induced production of mature IL-1β protein (Fig. 6c). We hypothesized that cell death might be required for the release of IL-1β, and indeed inhibition of death by GM-CSF (Fig. 6b, lower panel) correlated with diminished release of mature IL-1β (Fig. 6c). Furthermore, corresponding to GM-CSF inhibiting IL-21-mediated cDC death by inhibiting BIM expression^16^, we observed markedly decreased IL-21-induced apoptosis in the absence of BIM, which is encoded by the *Bcl2l11* gene (ref. 16 and Fig. 6d), and whereas IL-21-induced *Il1b* expression was not significantly altered in BIM-deficient cDCs (Fig. 6e), IL-21-induced IL-1β production was essentially abolished (Fig. 6f). In contrast, LPS+ATP-mediated apoptosis (Fig. 6d) and IL-1β production (Fig. 6f) were not dependent on BIM. These data indicate that IL-21 uses a distinctive, non-canonical pathway for IL-1β production.

### Apoptosis is critical for IL-21-mediated IL-1β production

**Figure 7:**
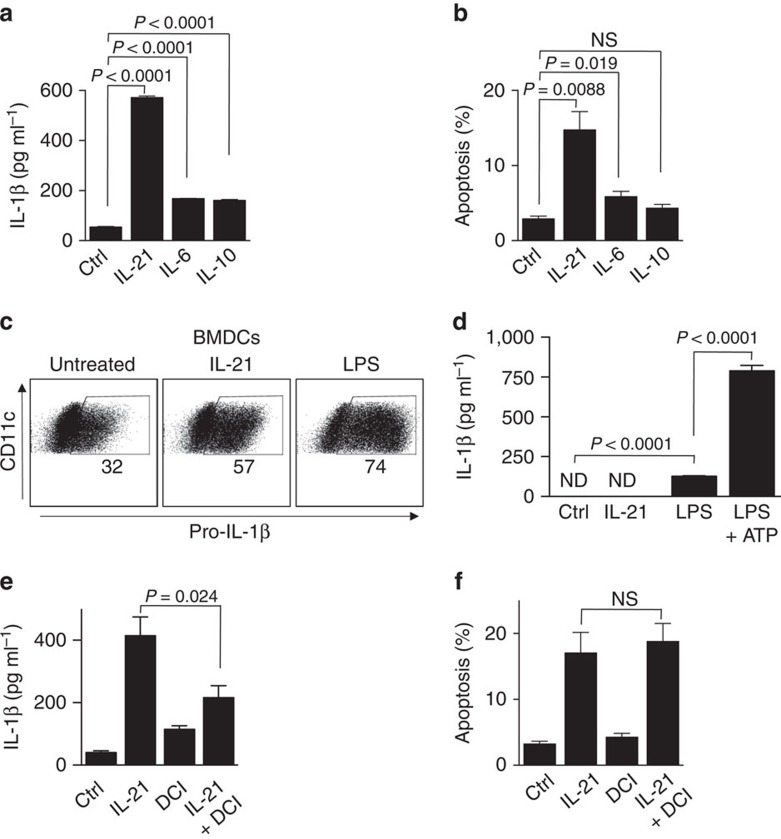
IL-21-induced IL-1β by cDCs is cell-death dependent. (**a**,**b**) cDCs were treated with 100 ng ml^−1^ IL-21, IL-6, or IL-10 for 24 h and IL-1β protein (**a**) and % apoptosis (**b**) determined. Data are from three experiments; error bars are means±s.e.m. In **b**, NS, *P*=0.076. (**c**) BMDCs were rested without GM-CSF for 16 h, treated with IL-21 or LPS for 4 h, and intracellular pro-IL-1β determined by flow cytometry. BMDCs were gated as CD11c^hi^ cells. Data are representative of two experiments with a total of six samples. (**d**) BMDCs were treated for 24 h with IL-21 or LPS±5 mM ATP added in the final 1 h, and IL-1β protein determined. Data are representative of three experiments with a total of eight individual samples; error bars are technical duplicates of the representative experiment. N.D., not detectable. (**e**,**f**) cDCs were treated with 20 μM of DCI for 1 h, then with IL-21 for 24 h. IL-1β protein production (**e**) and the % apoptotic cells was determined (**f**). Data are from four experiments; error bars are means±s.e.m. In **f**, NS, *P*=0.69. In Figure 6, IL-1β production in the culture supernatant was determined by ELISA. Pro-IL-1β induced by IL-21 in the culture supernatant was minimal, based on a pro-IL-1β-specific ELISA. Statistical analysis was performed by Student’s *t*-test.

We next compared the effect of IL-21 to those of IL-6 and IL-10, which also activated STAT3 and could induce pro-IL1β (Fig. 2a,b). IL-21 appeared somewhat distinctive, as IL-1β production and cDC death were only slightly induced by IL-6 and IL-10 (Fig. 7a,b), even though these cytokines were more similar to IL-21 in their induction of pro-IL-1β (Fig. 2a,b). These results underscore the distinctive ability of IL-21 to potently induce apoptosis and the production of mature IL-1β in cDCs. Despite its apoptotic effect on cDCs, IL-21 does not diminish the viability of BMDCs^16^, and although IL-21-induced pro-IL-1β expression in BMDCs (Fig. 7c), it did not induce the production of mature IL-1β, in contrast to its potent induction by LPS+ATP (Fig. 7d). cDC death may result in the induction of serine protease activity, and previous studies suggested that pro-IL-1β can also be processed by serine proteases^25^. We thus investigated the role of serine proteases in IL-21-mediated IL-1β expression by treating cDCs with a general serine protease inhibitor, 3,4 dichloroisocoumarin^26^ before IL-21. This inhibitor substantially inhibited IL-21-induced IL-1β production (Fig. 7e) but did not affect IL-21-induced apoptosis (Fig. 7f). Thus, activation of serine protease(s) is not required for IL-21-mediated cell death but rather occurs in association with or following cell death. These data demonstrated that IL-21-STAT3 axis induces a non-canonical, cell death- and serine protease-dependent pathway for IL-1β production in cDCs.

### IL-21-mediated IL-1β in the pathologic response to PVM

**Figure 8:**
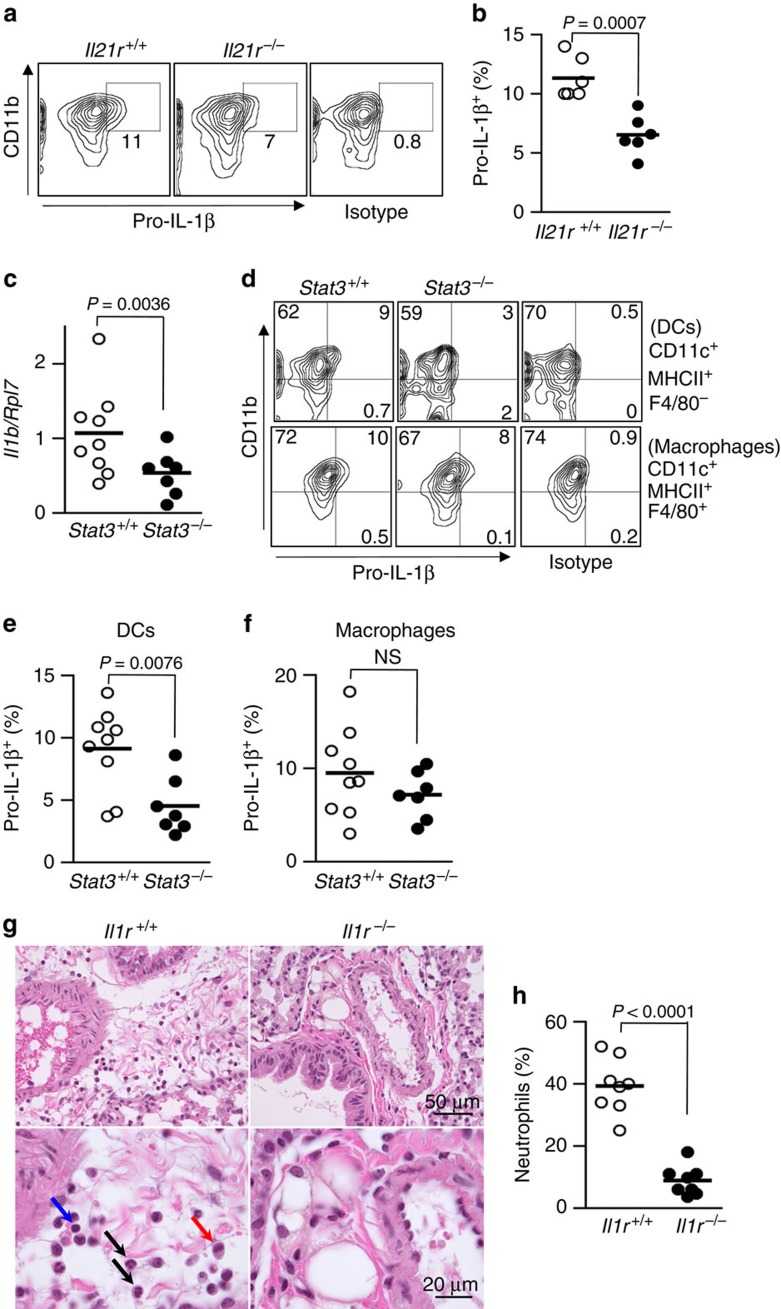
IL-21-STAT3 signaling contributes to the expression of IL-1β during PVM infection. (**a**,**b**) *Il21r*^+/+^ and *Il21r*^−/−^ mice were infected with 60 plaque-forming unit PVM for 6 days. Expression of intracellular pro-IL-1β in DCs (CD11c^+^MHCII^+^F4/80^−^) was determined by flow cytometry. Shown are representative plots (**a**) and summary of data from multiple animals (**b**). Data from two experiments (*n*=6). (**c**–**f**) *Stat3*^+/+^ and *Stat3*^−/−^ mice were infected with 60 plaque-forming unit PVM for 6 days. (**c**) RNA was isolated from lungs and *Il1b* mRNA expression was analysed by RT–PCR. (**d**–**f**) Expression of intracellular pro-IL-1β in DCs (CD11c^+^MHCII^+^F4/80^−^) (**b**,**c**) and macrophages (CD11c^+^MHCII^+^F4/80^+^) (**b**,**d**) was determined by flow cytometry. Shown are representative plots (**d**) and summary of data of DCs (**e**) and macrophages (**f**) from multiple animals. Data are from two experiments (*Stat3*^+/+^, *n*=9; *Stat3*^−/−^, *n*=7). In **f**, NS, *P*=0.26. (**g**,**h**) *Il1r*^+/+^ and *Il1r*^−/−^ mice were infected with PVM as in **a**. (**g**) Representative lung sections (stained with H&E) from *Il1r*^+/+^ and *Il1r*^−/−^ mice after PVM infection for 6 days. Top: Compared with *Il1r*^−/−^ mice, *Il1r*^+/+^ lungs showing severe oedema at day 6 after PVM infection. Bottom: higher magnification; neutrophils (black arrows), macrophages (red arrow) and lymphocytes (blue arrow). (**h**) Percent neutrophils (CD11b^hi^ Ly6G^+^, determined by flow cytometry) in lungs after PVM infection for 6 days. Data are from two experiments (total of eight mice per group). Statistical analysis was performed by Student’s *t*-test.

IL-21 promotes the pathological immune response of PVM but the mechanism is unclear. We found less *Il1b* mRNA in the lungs of *Il21r*^−/−^ mice than in wild-type (WT) mice after infection with PVM^27^. Correspondingly, cDCs from *Il21r*^−/−^ mice had lower expression of pro-IL-1β after PVM infection (Fig. 8a,b). Consistent with the role of STAT3 in IL-21-mediated IL-1β expression, *Il1b* mRNA was also lower in lungs from *Stat3*^−/−^ than from WT mice after PVM infection (Fig. 8c). The absence of STAT3 significantly lowered pro-IL-1β expression in cDCs (Fig. 8d,e), whereas pro-IL-1β production tended to be lower in macrophages, although the decrease was not statistically significant (Fig. 8d,f), analogous to the *in vitro* data (Fig. 2f). Moreover, *Il1r*-deficient mice had less perivascular oedema and fewer immune cells (Fig. 8g, right versus left panel), and particularly fewer neutrophils (Fig. 8h) in the lung. These results indicate that IL-1β is a key mediator of inflammation, and collectively indicate the importance of the IL-21-STAT3-IL-β axis in the pathological immune response to PVM infection.

## Discussion

In this study, we have identified an unanticipated role of IL-21 in the regulation of IL-1β expression in cDCs and provide an example by which a type 1 cytokine regulates IL-1β. cDCs develop from precursor cells that are distinct from those giving rise to monocyte-derived DCs^28^. Whereas monocyte-derived DCs are important for inflammatory responses, cDCs play critical roles in triggering immune responses as well as maintaining immune self-tolerance through their interaction with T cells and the production of cytokines and chemokines^29^. The molecular mechanisms controlling IL-1β in cDCs previously have not been studied, and we found that in addition to using a canonical pathway (for example, for LPS) that is dependent on NF-κB for *Il1b* gene expression and the caspase-1-ASC-NLRP3 inflammasome for pro-IL-1β processing, cDCs can also use an alternative STAT3-dependent pathway for IL-21-induced *Il1b* gene expression. STAT3 bound at the *Il1b* promoter and 5′ upstream region, suggesting that it directly regulates *Il1b* mRNA expression. Interestingly, this IL-21-STAT3 pathway for regulating IL-1β seems to be cDC-specific, as IL-21 did not induce *Il1b* mRNA in CD4^+^ T cells even though it strongly activated STAT3 in these cells. Indeed, IL-21-activated STAT3 bound mostly to non-overlapping sites in cDCs and CD4^+^ T cells, and these different binding patterns likely account for the cell type-specific gene regulation by IL-21 (for example, induction of *Il1b* in cDCs versus *Il21* in CD4^+^ T cells). We further show that this distinctive STAT3 binding pattern correlates with the differential presence of marks for active enhancer landscapes in the different cell types, suggesting that epigenetic regulation is involved in the control of cell type-specific actions of IL-21. Previous studies have identified cell type-specific enhancer landscapes in Th1 and Th2 cells^30^, but our results reveal that differential enhancer landscapes in cDCs and CD4^+^ T cells determine the cell type-specific effect of IL-21.

Strikingly, we found that IL-21 not only induced *Il1b* mRNA expression in cDCs but also triggered pro-IL-1β processing and release of mature IL-1β, and that a second, inflammasome-activating signal was not required. Indeed, IL-21 acted in the absence of either caspase-1 or caspase-8, but IL-21-induced IL-1β production was strictly dependent on IL-21-mediated cell death, which requires STAT3-mediated induction of BIM^16^. Interestingly, the actions of IL-21 are distinctive because IL-6 and IL-10 minimally induce cDC death even though these cytokines also activate STAT3.

In this study, we have found that IL-21-mediated mature IL-1β production and release from cDCs are strictly dependent on cell death. Our data indicate that this process requires the activation of serine protease(s), which likely occurs in association with or following IL-21-induced cell death. Interestingly, previous studies have shown that serine proteases such as cathepsin G, elastase and proteinase 3 can cleave pro-IL-1β into active IL-1β in neutrophils^25^, which is responsible for caspase-1-independent IL-1β production during acute arthritis induction in mice^31^. Additional work is required to determine the *in vivo* importance of the novel IL-21-mediated, serine protease-dependent IL-1β production pathway we have identified as well as the importance of the induction of *IL1B* mRNA by human DCs.

Our studies establish a novel crosstalk between IL-21 and IL-1β, and this crosstalk may contribute to the uncontrolled inflammatory responses during PVM-induced lung disease. Additional work is required to determine the importance of this mechanism versus that of the canonical major NF-κB-inflammasome-dependent pathway in lung DCs and macrophages for the production of IL-1β during PVM infection. Conceivably, the pathway we describe might also contribute to autoimmune diseases where high levels of IL-21 and IL-1β can be detected^7^,^12^. These studies significantly expand our understanding of the actions of IL-21 in cDCs, with IL-21 both promoting immune tolerance through the induction of apoptosis of cDCs and also promoting inflammatory responses via its induction of IL-1β.

## Methods

### Mice and reagents

C57BL/6, 129X1/SvJ, *Bcl2l11*^−/−^, *Il1r*^*−/−*^ and CD11c-*Cre* mice were from The Jackson Laboratory. *Stat3*-deficient DCs were generated by crossing *Stat3*-floxed mice with CD11c-*Cre* mice^16^. *Il21r*^*−/−*^ mice have been described^10^. *Ripk3* and *Casp8* double-deficient mice have been described^24^. *Casp1*^−/−^ mice were from The Jackson Laboratory or from Dr Richard Flavell, Yale University^32^. *Nlrp3*^−/−^ and *Pycard*^−/−^ mice were from Dr Vishva Dixit, Genentech^33^,^34^. Both male and female mice from 7 to 12 weeks old were used. All protocols were approved by the NHLBI Animal Care and Use Committee and followed NIH guidelines for using animals in intramural research. MLN120B was provided by Dr Ulrich Siebenlist, NIH.

### Isolation of splenic DCs

Mouse spleens were injected with 1 ml of 1 mg ml^−1^ collagenase D and 20 μg ml^−1^ DNase I (Roche), cut and incubated in collagenase solution at 37 °C for 20 min. After passage through a cell strainer, red blood cells were lysed using ACK lysis buffer and the remaining cells were incubated with Fc block (BD Biosciences) at 4 °C for 10 min. CD11c^+^ cells were positively selected with CD11c microbeads (Miltenyi Biotec). Purity of CD11c^+^ DCs was 93–95%.

### Isolation of human peripheral blood CD1c^+^ cDCs

Buffy coats from healthy volunteers were separated by gradient centrifugation using lymphocyte separation medium. Mononuclear cells were negatively selected using CD19 microbeads to remove the CD1c^+^ B cells, followed by positive selection with CD1c microbeads (Miltenyi Biotech) to enrich for CD1c^+^ cDCs (purity was >90%).

### Generation of BMDCs and BMMs

Bone marrow cells from mouse femurs and tibias were cultured for 8 days in RPMI-1640 medium containing 10% FBS, 200 μM L-glutamine, 10 IU ml^−1^ penicillin, 100 μg ml^−1^ streptomycin, 55 μM β-mercaptoethanol and 20 ng ml^−1^ GM-CSF (for BMDCs) or macrophage colony-stimulating factor (for BMMs) (Peprotech), with medium changed every 3 days. Cells were >90% pure.

### FACS analysis of phosphorylated STAT proteins

Cells were stained with mouse CD11c (N418, 1/200 and eBioscience), F4/80 (BM8, 1/200, Biolegend), or TCR-β (H57-597, 1/200, Biolegend) antibodies at 37 °C for 10 min, stimulated with cytokines for 15 min, fixed with 2% paraformaldehyde at 37 °C for 10 min, permeabilized in 90% ice-cold methanol for 30 min, washed twice with FACS buffer (PBS with 1% FBS), stained with pSTAT3 (Y705)-PE or pSTAT3 (Y705)-Alexa Fluor 647 antibodies (4/P-STAT3, 1/25 and BD Biosciences) at RT for 30 min, and analysed on a FACSCanto II (Becton Dickinson).

### RNA analysis

Total RNA was isolated using the RNeasy Plus Mini Kit (Qiagen). First-strand complementary DNAs were synthesized using the Omniscript reverse transcription kit (Qiagen) and oligo(dT). Quantitative reverse transcription–PCR was performed on a 7900H sequence detection system (Applied Biosystems). Real-time primers and TaqMan probes were from Applied Biosystems. Expression was normalized to mouse *Rpl7* or human *ACTB*.

### Apoptosis studies

Splenic DCs were rested 1 h, stimulated with different cytokines for 20–24 h, and apoptosis was analysed by Annexin V and 7-AAD staining (BD Biosciences). For some experiments, the cells were incubated with 20 μM of serine protease inhibitor 3,4 dichloroisocoumarin for 1 h before cytokine stimulation.

### Bioactivity assay of mature IL-1β

Naïve CD4^+^ T cells were isolated from WT and *Il1r*^*−/−*^ mice and differentiated into Th17 cells with 5 μg ml^−1^ plate-bound anti-CD3, 2 μg ml^−1^ soluble anti-CD28, 10 ng ml^−1^ IL-6, 2 ng ml^−1^ TGF-β, 10 μg ml^−1^ anti-IFN-γ, 10 μg ml^−1^ anti-IL-4 for 2 days. cDC supernatants from un-stimulated or IL-21-stimulated cultures were added to the cells and incubated for 2 days, without or with the addition of 10 μg ml^−1^ anti-IL-1β. Expression of IL-2Rα on Th17 cells was determined by flow cytometry. Amounts of mature IL-1β in the supernatants were determined by a standard curve (0–10 ng ml^−1^ recombinant IL-1β)

### PVM infection and lung cell preparation

Virus stock (PVM strain J3666) was prepared as previously described^35^. Mice were anaesthetised briefly by ketamine/xylazine and inoculated intranasally with 60 plaque-forming unit PVM in 50 μl PBS. For flow cytometry analysis, lung tissue was minced into small pieces using a razor blade and digested in a solution containing 1 mg ml^−1^ collagenase D and 200 μg ml^−1^ DNase I (Roche) for 30 min at 37 °C. Digested tissue was then pushed through a cell strainer with a syringe. Cells were centrifuged, and RBCs were lysed with ACK, followed by two washes with complete RPMI-1640 medium. For RNA analysis, lung tissue was homogenized in TRIzol (Invitrogen) followed by RNA cleanup with the RNeasy kit (Qiagen).

### ChIP-Seq analysis

DCs were rested 1 h and treated with IL-21 for 1 h; CD4^+^ T cells were pre-activated with 5 μg ml^−1^ plate-bound anti-CD3+2 μg ml^−1^ soluble anti-CD28 for 3 days, washed, rested for 16 h and treated with IL-21 for 1 h. Cells were fixed with 1% formaldehyde at 37 °C for 10 min. Chromatin from 2 × 10^7^ cells was sonicated for 20 cycles of 12 s on per 48 s off, into 200–500 bp fragments, DNA ends were repaired using polynucleotide kinase and Klenow enzyme and treated with Taq polymerase to generate a protruding 3′ ‘A’ nucleotide. DNA fragments of ∼250–450 bp were purified from agarose gels and used for cluster generation and sequencing. Antibodies against STAT3 (13–7,000, 4 μg, Invitrogen), H3K4me1 (ab8895, 3 μg, Abcam), H3K4me3 (17–614, 3 μg, Millipore), H3K27me3 (17–622, 3 μg, Millipore), H3K27ac (ab4729, 3 μg, Abcam) were used.

PCR products were barcoded (indexed) and sequenced on an Illumina HiSeq 2000 platform. Sequenced reads (50 bp, single end) were obtained with the Illumina CASAVA1.8 pipeline and mapped to the mouse genome (NCBI37/mm9, July 2007) using Bowtie 0.12.9^36^. Only uniquely mapped reads were retained. To eliminate bias caused by PCR amplification, only non-redundant reads were retained and analysed. The mapped outputs were converted to browser-extensible data files, which were then converted to binary tiled data files (TDFs) using IGVTools 2.3.32 for viewing on the IGV browser (http://www.broadinstitute.org/igv/home). TDFs represent the average alignment or feature density for a specified window size across the genome. We mapped reads into non-overlapping 200 bp windows (for transcription factors STAT3) or 20 bp windows (for histone modifications such as H3K4me1 or H3K27ac) and the reads were shifted 100 bp from their 5′ starts to represent the center of the DNA fragment associated with the reads.

See Supplementary Table 2 for the summary of ChIP-Seq libraries.

### RNA-Seq analysis

Total mouse splenic CD4^+^ T cells were pre-activated with plate-bound anti-CD3+anti-CD28 for 3 days, washed and rested overnight, and not stimulated or stimulated with IL-21 for 4 h. Total RNA was isolated, and 5 μg per sample were used for mRNA purification using Dynal oligo(dT) beads (Invitrogen). Double-stranded complementary DNA was synthesized with random hexamer primers, SuperScript II, DNA polymerase I and T4 DNA polymerase (Invitrogen) and fragmented by Bioraptor. After end repair (Epicentre DNA END-Repair kit) and the addition of an ‘A’ nucleotide to the 3′ ends with Taq DNA polymerase, two mixed Solexa adaptors (Illumina) were ligated to DNA ends using T4 DNA ligase (New England Biolabs). Fragments (250–500 bp) were isolated on 2% E-Gels (Invitrogen) and amplified for 18 cycles using PE 1.0 and 2.0 primers (Illumina) and Phusion High Fidelity PCR Master Mix (New England Biolabs). PCR products were run on a gel, and 250–500 bp fragments were purified. Final PCR products were sequenced on an Illumina HiSeq 2000 platform. Sequenced reads (50 bp, single end) were mapped to the mouse genome (NCBI37/mm9, July 2007) using Bowtie 0.12.9, and only the reads that mapped on exons of each RefSeq gene were measured and normalized using reads per kilobase per million mapped reads. The analyses of differential gene expression were performed using the R package edgeR. See Supplementary Table 2 for the summary of RNA-Seq libraries.

### ChIP-Seq peaks and binding distribution

MACS 1.4.2 (ref. 37) was used to call binding sites (peaks) relative to control libraries. The *P* value threshold was set as 1 × 10^−5^ and effective genome size was set as 1.87 × 10^9^. Only non-redundant reads were analysed for peak calling. A transcription factor (such as STAT3) was considered as ‘bound’ to genes if peaks were within 5 kb upstream of the transcription start site and anywhere across the gene body.

### Binding intensity distribution

To compute the binding distribution of transcription factors or histones across selected genomic regions (such as STAT3 peaks), we chose±3 kb regions around either transcription start site or peak summits and divided the regions into bins of 20-bp window size. Reads (or tags) that fell into each bin were counted and normalized by library size. The normalized read density was plotted using Gnuplot 4.6. Heatmap of binding intensity was generated based on K-means clustering and plotted using seqMiner 1.3.3e^38^.

### Statistical analysis

Statistical comparison between samples was performed by unpaired Student’s *t*-test, *P*<0.05 is considered as statistically significant. NS, not statistically significant.

## Additional information

**Accession codes:** RNA-Seq and ChIP-Seq data are accessible through GEO SuperSeries accession code GSE61677.

**How to cite this article:** Wan, C.-K. *et al.* IL-21-mediated non-canonical pathway for IL-1β production in conventional dendritic cells. *Nat. Commun.* 6:7988 doi: 10.1038/ncomms8988 (2015).

## Supplementary Material

Supplementary Figures and Tables Supplementary Figures 1-7 and Supplementary Tables 1-2

Supplementary Data 1 Genes regulated in cDCs by LPS (a), IL-21 (b), IL-10 (c), IL-21 but not IL-10 (d), IL-10 but not IL-21 (e), and both IL-21 and IL-10 (f).

Supplementary Data 2 Shown are genes regulated by LPS in cDCs whose expression is altered by IL-21 (a), IL-10 (b), IL-21 but not IL-10 (c), IL-10 but not IL-21 (d), or both IL-21 and IL-10 (e).

Supplementary Data 3 IL-21-induced STAT3 peaks in pre-activated CD4+ T cells (a) and cDCs (b).
