# Supplementary material for: IL-21-mediated non-canonical pathway for IL-1β production in conventional dendritic cells
Source: Nat Commun. 2015 Aug 13;6:7988. doi: 10.1038/ncomms8988 (PMC4555999; doi:10.1038/ncomms8988)
Supplement: Supplementary Figures and Tables — Supplementary Figures 1-7 and Supplementary Tables 1-2 [file ncomms8988-s1.pdf]

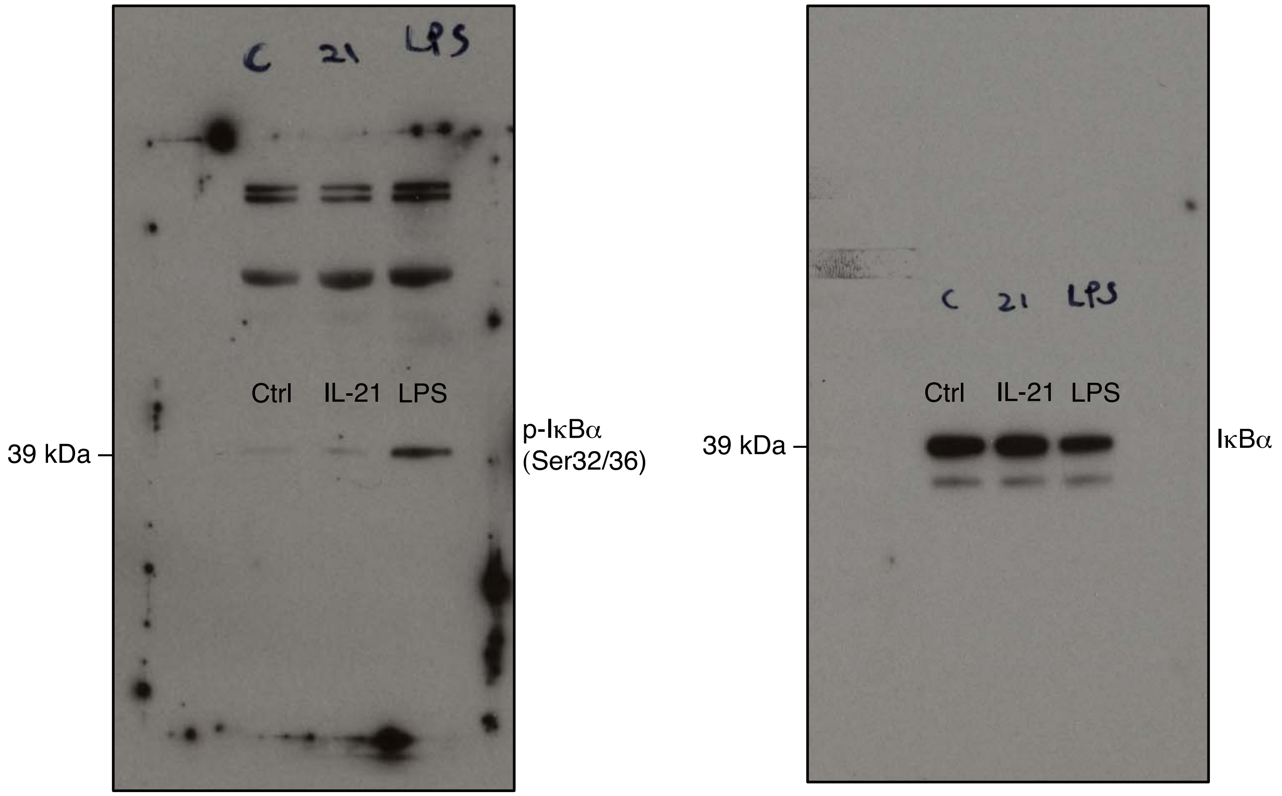

Supplementary Figure 1. LPS but not IL-21 stimulation induces IκBα phosphorylation. cDCs were rested 1 h, stimulated with 100 ng ml^{-1} IL-21 or LPS for 30 min, and the expression of phosphorylated (left) and total (right) IκBα was determined. Shown is one of two similar experiments. See also Figure 1e.

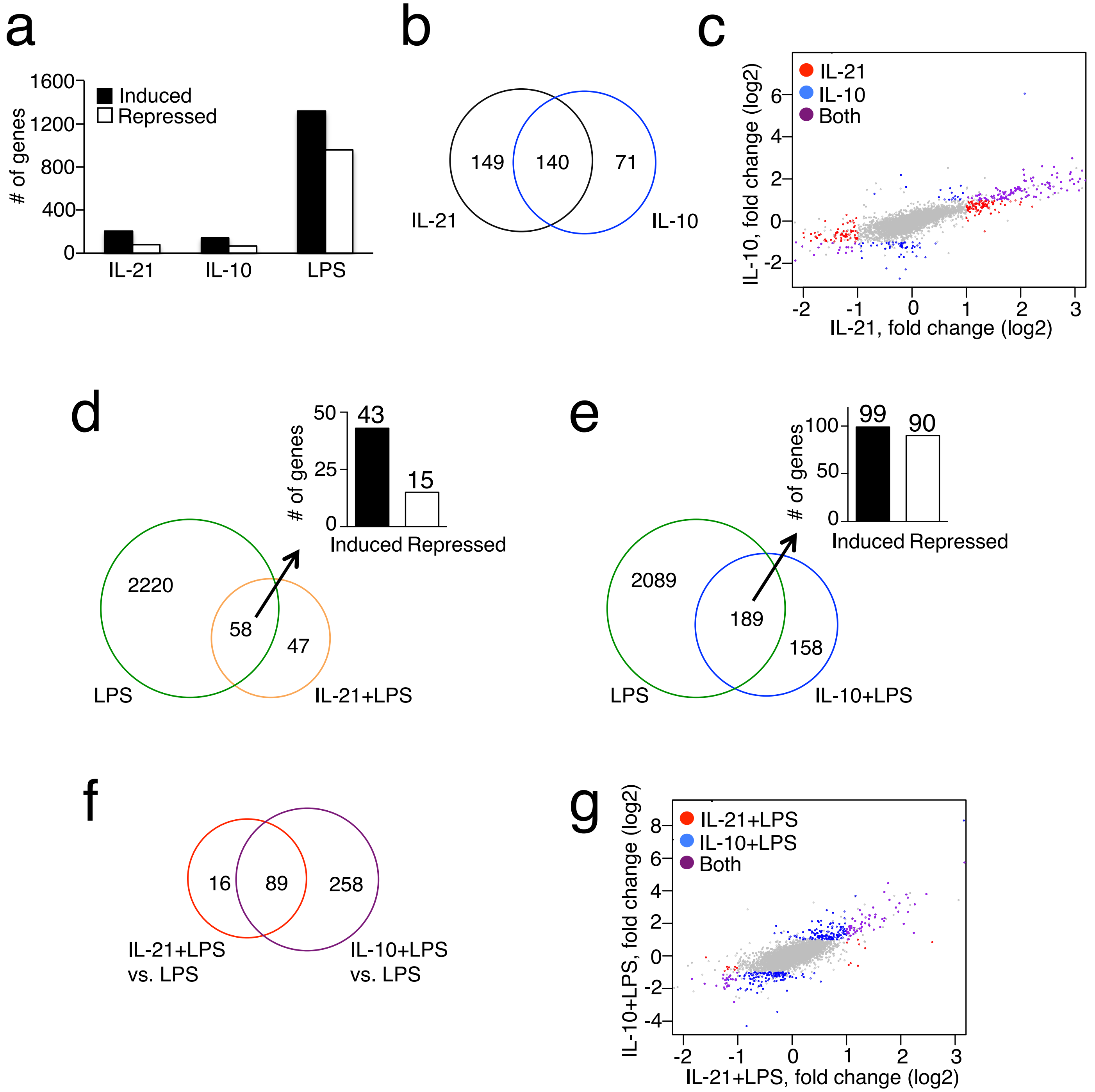

Supplementary Figure 2. RNA-Seq analysis of cDCs after cytokines and LPS stimulation. (**a-c**) cDCs were stimulated with 100 ng ml^{-1} of IL-21, IL-10 or LPS for 4 h. (**a**) Numbers of genes that were induced or repressed by IL-21, IL-10, or LPS. Venn diagram (**b**) and dot plot (**c**) showing genes that were regulated by IL-21, IL-10, or both cytokines. (**d-g**) cDCs were stimulated with LPS with or without pre-treatment of IL-21 or IL-10 1 h prior LPS stimulation. (**d,e**) Venn diagrams showing numbers of LPS-regulated genes, which their expression was affected by IL-21 (**d**) or IL-10 (**e**). Histograms show the numbers of genes that were induced or repressed by LPS in cells not pretreated versus pretreated with IL-21 (**d**) or IL-10 (**e**). (**f,g**) Venn diagram (**f**) and dot plot (**g**) showing the numbers of LPS-regulated genes whose expression was affected by IL-21, IL-10, or both cytokines. (**a-g**) Data shown are representative of 2 independent experiments.

a

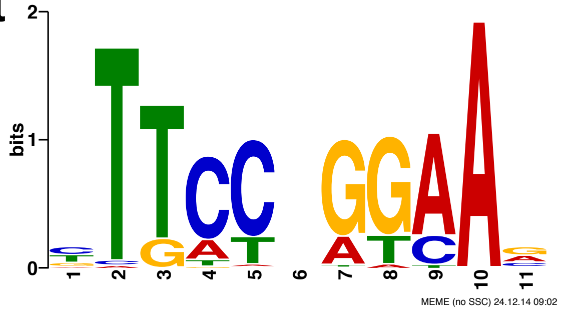

Top STAT3 binding motif in CD4⁺ T cells.
384/500 sites, E-value: 1.6e-293

b

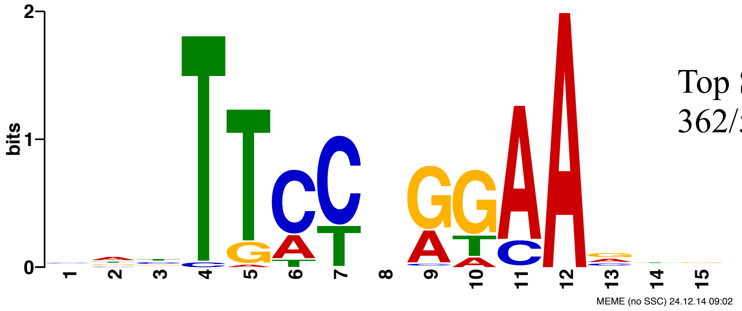

Top STAT3 binding motif in cDCs.
362/500 sites, E-value: 3.8e-209

Supplementary Figure 3. Motif analysis of STAT3 binding sites in IL-21-stimulated CD4⁺ T cells (**a**) and cDCs (**b**). Canonical gamma interferon-activated site (GAS) motif was identified as the dominant motif in both cell types after IL-21 stimulation.

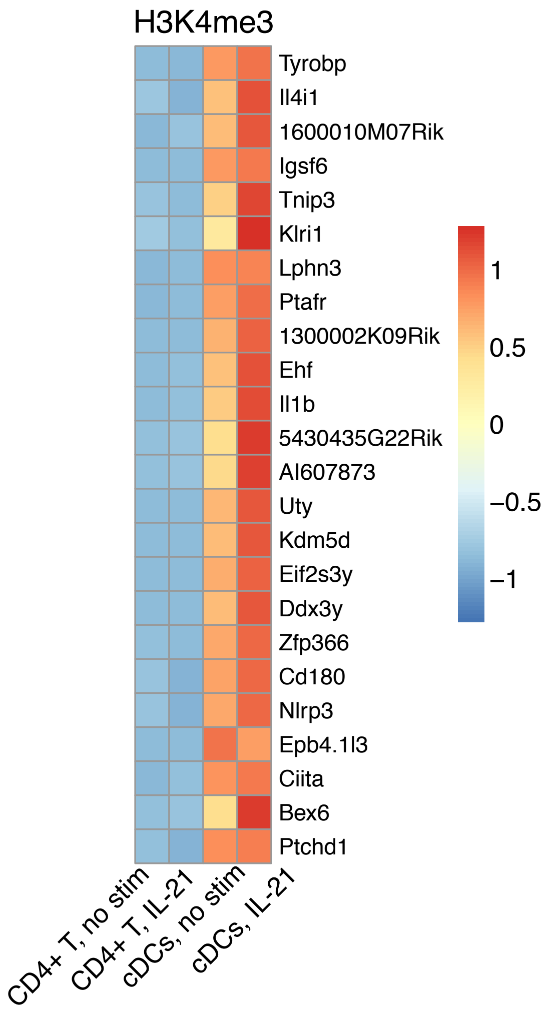

Supplementary Figure 4. Genes with enhanced IL-21-induced H3K4me3 in cDCs than in $CD4^+$ T cells. *Il1b* is one of only 24 genes with high H3K4me3 near the promoter in cDCs but not $CD4^+$ T cells.

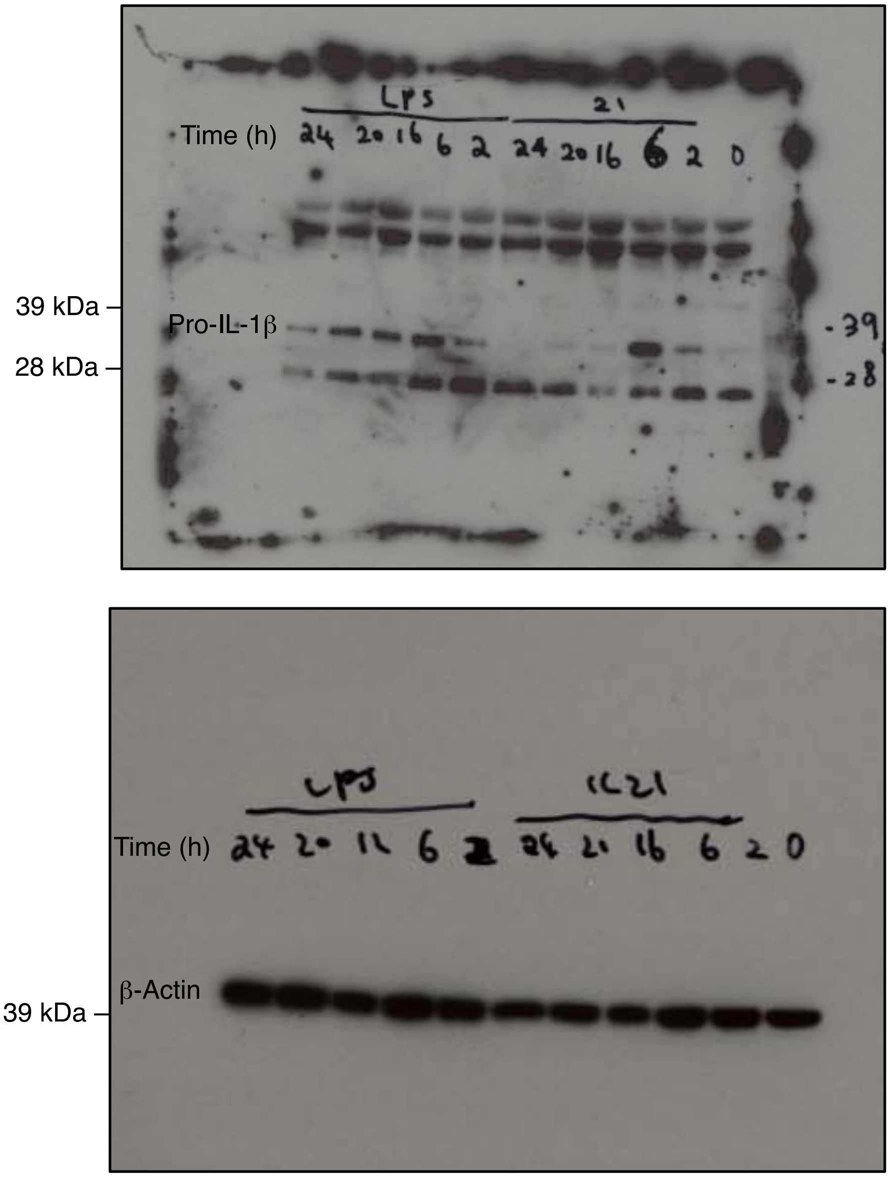

Supplementary Figure 5. IL-21 and LPS induce pro-IL-1β expression with different kinetics. cDCs were rested 1 h, treated with 100 ng ml^{-1} IL-21 or LPS at the indicated time points, and intracellular pro-IL-1β expression (upper) was determined. β-actin (bottom) was used as control. Shown is one of two similar experiments. See also Figure 5a.

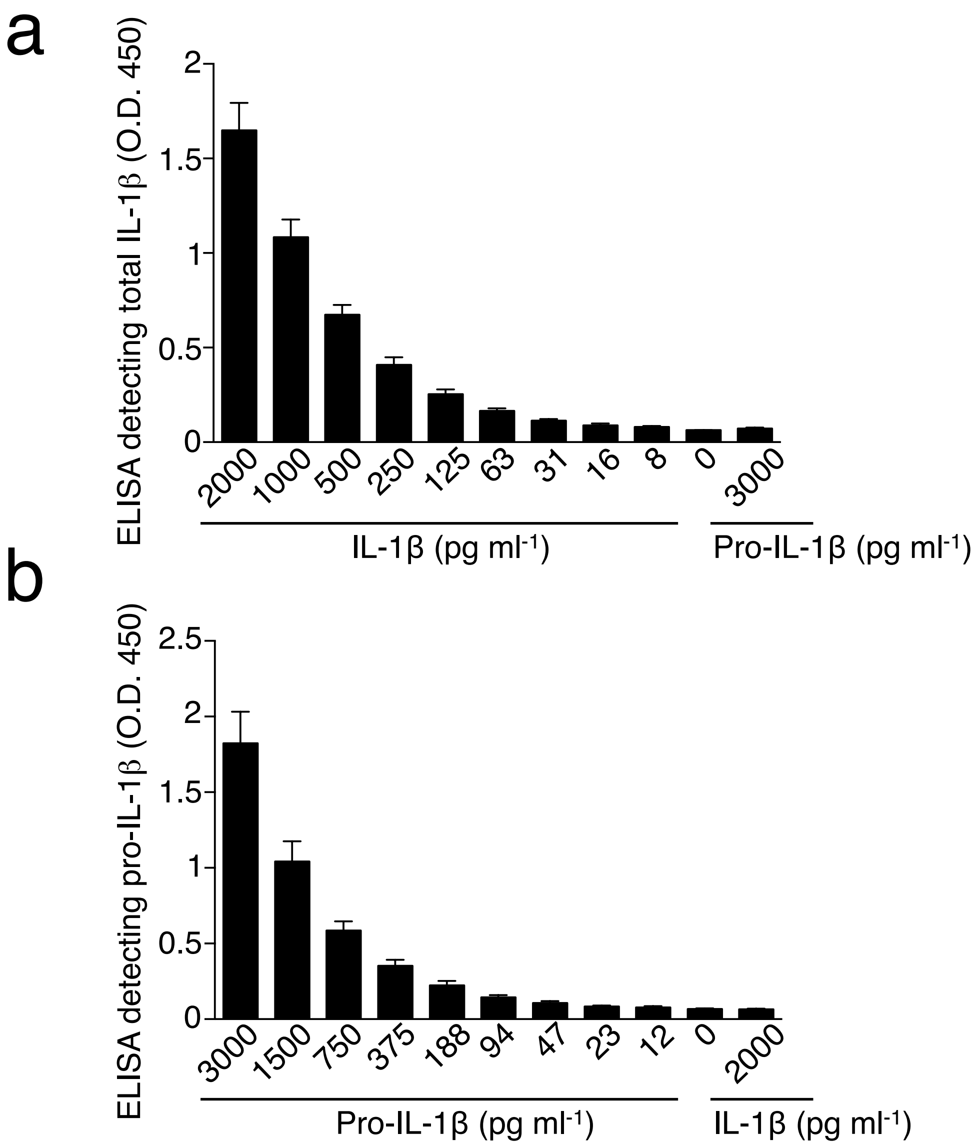

Supplementary Figure 6. Antibodies recognizing mature IL-1β in ELISA do not cross-react with pro-IL-1β. (**a**) Serial dilutions of recombinant IL-1β (8-2000 pg ml^{-1}) and 3000 pg ml^{-1} of recombinant pro-IL-1β was tested with ELISA detecting IL-1β, signal intensity was determined by O.D. 450. (**b**) Serial dilutions of recombinant pro-IL-1β (12-3000 pg ml^{-1}) and 2000 pg ml^{-1}of recombinant IL-1β was tested with ELISA detecting pro-IL-1β, signal intensity was determined by O.D. 450. Shown are results combined from 3 independent experiments, error bars are means ± SEM.

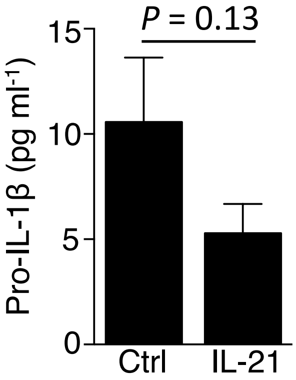

Supplementary Figure 7. IL-21 does not induce secretion of pro-IL-1β. cDCs were rested 1 h, stimulated with IL-21 for 24 h, and the amounts of pro-IL-1β in the culture supernatants were determined by ELISA. Shown are combined results of 3 independent experiments; error bars are means ± SEM. Statistical analysis was performed by Student's *t*-test.

***Il1b* GAS1**	**chr2:129196856-129197256**
1-50	AACCACTGCAGGGTTTGTTGTCCAACTTGTTTTCCCTCCCTTGTTTTATA
51-100	GTCCTAAAAAGCAGAAGTGAAGAGCTGTGAAATTTTCCCTTGGGTTAACT
101-150	GATTTCACAACAAAACTGAAGGGTGGGGGAGGGGGGAGAAGCTTGATGGG
151-200	AATTC**TTAGGGGAA**GAGGCTATTGC**TACCCTGAA**ATAATTTCTAA**TCCCT**
201-250	**GGAA**GTCAAGGGGTGGCAGATAGTGACACATTCGCAAGTGTGTCATCGTG
251-300	GTGGAAATGGGCATTATTTCCCCCTGGACAATTGTGCAGATGGTGTCAAT
301-350	ATTAAAAATATTGGGTTTTCCTGGGTTAGCTGTACATTTTAAGTCAGGAT
351-400	GTGCGGAACAAAGGTAGGCACGTAGATGCACACCCAGAAGTGTGTCACCT

***Il1b* GAS2**	**chr2:129199054-129199454**
1-50	GTGACTTGGGCTCCAAATTTCCTCCCCTTGTCGTTATAGAGCAGGCAAGT
51-100	TGCACAACATGTATATCAAACCCTGTCTCTCAGACCACACTTCCTTTTTG
101-150	GTCCCCAATGAGTAGAAGAAATAACTCACTATAGAAATTGTTATGCTGGG
151-200	GGTGTGGCATAGTGATGACGATACTGATGATGATGTTGGCAAAGGAAATG
201-250	AACAAAGCAAAAGAGGAAGTCGGCAAAGCCAGGATGGTGACGGGCACTCT
251-300	AGCT**TTTGGGGAA**CTTTTCAGCTCTTGACTCACTCAGCACTTTTAAGCTG
301-350	TAAGGTTCCCTCTGTTCTGACATCTCA**TTCCTCCAA**GATGGAGGCAGGCA
351-400	T**TTCTTCAAA**GCTGAAAGTGCCTTGCTACCTGTTTTACTTACTTTCTATT

***Il1b* GAS3**	**chr2:129206523-129206923**
1-50	AGGAATTCTGCTCCTGTGTTTCTACACTGTGCTGCTGACATGGGGGCCAA
51-100	CTCCATTCCATACCTTTCTTCTTTGCCCTTCAGTGGGTGACATG**TTGTGT**
101-150	**GAA**TTTGTTTCTTTCTCTGTCTCTGGGGGCAACACTGGGACTTTCCAAAT
151-200	GAGTCGTGGGTTGTGTTTCTTCCCCTTTTCCGAGCTTCTTCCTTATTCCG
201-250	ATTCACTTCCTCACCTGTGACTGGCAGCCTTGGTTATGGATGCCCTTTTG
251-300	TTTTCTCCTTCTCTGCTTTAAAAAAAAATCTCTTCAGGGTTTATGCCTTT
301-350	GGAATAATGTTTTTTCTTTATGGACTTGGGGCCCTCCCATCTATGATAAA
351-400	CTACTTCTAACATGTCTGATCTGCCTATTTCATCTCTCTGGTTTTTACTT

***Il21* GAS**	**chr3:37130101-37130501**
1-50	ACACACTGAGAAATGTTCTCAC**TTATTGGAA**ATGTCCTTTCAATAAGTTT
51-100	GAATGAGCTACTTTCAGTTTAGAGGCCACTCATTTGAAAGGACCTAGTAA
101-150	ACACAAGAATGATCATGTGCAGAGCTGGAGAGTGAACTGTGGAGCAGAGG
151-200	GGCGGCGTGACTCACTCACTTTCTAAACTCTTACGCTGTCATTTACCATA
201-250	ATTCTC**TTCTTTGAG**TGTCTCTGTGTGGTGCCCTCATTACTGGCAATACT
251-300	CTCCTTGTTTTCCCGATTTATGCAATTAAAGGCCTAGCTGTGCGAGCATT
301-350	TCCCCAGTTTCATTTACATTGGTACCAAGCTGAAGACCAGAATAAAGATG
351-400	TTCTCAGGGCAGAGACAGCAGAAGCCCCTCCACACTGAATGCCAAGCCAC

Supplementary Table 1. STAT3-bound GAS motifs at *Il1b* and *Il21* loci

Samples	Descriptions	source name	molecule	Raw Reads	Mapped Reads	Mappability (%)	Non-Dup Reads
Sample 1	WT CD4 T -IL21 STAT3	Pre-activated CD4+ T cells	genomic DNA	52459212	29318548	55.89	3064636
Sample 2	WT CD4 T +IL21 STAT3	Pre-activated CD4+ T cells	genomic DNA	53246033	34856333	65.46	4299879
Sample 3	WT CD4 T -IL21 H3K4me3	Pre-activated CD4+ T cells	genomic DNA	25176494	19773538	78.54	9938066
Sample 4	WT CD4 T +IL21 H3K4me3	Pre-activated CD4+ T cells	genomic DNA	22298910	17210565	77.18	11120304
Sample 5	WT CD4 T -IL21 H3K27me3	Pre-activated CD4+ T cells	genomic DNA	46973935	36822054	78.39	23301908
Sample 6	WT CD4 T +IL21 H3K27me3	Pre-activated CD4+ T cells	genomic DNA	41707447	32442386	77.79	22227675
Sample 7	WT CD4 T -IL21 H3K4me1	Pre-activated CD4+ T cells	genomic DNA	30716855	26475674	86.19	23715787
Sample 8	WT CD4 T +IL21 H3K4me1	Pre-activated CD4+ T cells	genomic DNA	29700046	25409258	85.55	22661636
Sample 9	WT CD4 T -IL21 H3K27ac	Pre-activated CD4+ T cells	genomic DNA	40216859	35874382	89.20	24378010
Sample 10	WT CD4 T +IL21 H3K27ac	Pre-activated CD4+ T cells	genomic DNA	39276670	34752929	88.48	24852829
Sample 11	WT DC T -IL21 STAT3	Splenic conventional DC	genomic DNA	36718461	25798330	70.26	7803557
Sample 12	WT DC T +IL21 STAT3	Splenic conventional DC	genomic DNA	35216560	25143231	71.40	14898280
Sample 13	WT DC -IL21 H3K4me3	Splenic conventional DC	genomic DNA	30196322	19326456	64.00	4619675
Sample 14	WT DC +IL21 H3K4me3	Splenic conventional DC	genomic DNA	11765527	9142362	77.70	5155878
Sample 15	WT DC -IL21 H3K27me3	Splenic conventional DC	genomic DNA	50675058	37974888	74.94	16695091
Sample 16	WT DC +IL21 H3K27me3	Splenic conventional DC	genomic DNA	43371825	31938973	73.64	13989743
Sample 17	WT DC -IL21 H3K4me1	Splenic conventional DC	genomic DNA	36503001	31111395	85.23	27312331
Sample 18	WT DC +IL21 H3K4me1	Splenic conventional DC	genomic DNA	42275343	35652301	84.33	32578563
Sample 19	WT DC -IL21 H3K27ac	Splenic conventional DC	genomic DNA	37833848	33439447	88.39	25931667
Sample 20	WT DC +IL21 H3K27ac	Splenic conventional DC	genomic DNA	37834047	33427687	88.35	26314709
Sample 21	RNASeq CD4 T untreated Rep1	Pre-activated CD4+ T cells	total RNA	47665163	33295358	69.85	*33295358*
Sample 22	RNASeq CD4 T +IL21 4h Rep1	Pre-activated CD4+ T cells	total RNA	54469530	37491069	68.83	*37491069*
Sample 23	RNASeq CD4 T untreated Rep2	Pre-activated CD4+ T cells	total RNA	45660899	31792719	69.63	*31792719*
Sample 24	RNASeq CD4 T +IL21 4h Rep2	Pre-activated CD4+ T cells	total RNA	54339602	37469602	68.95	*37469602*
Sample 25	RNASeq DCs un-stimulated	Splenic conventional DC	total RNA	59209672	53302010	90.02	*53302010*
Sample 26	RNASeq DCs LPS	Splenic conventional DC	total RNA	55348231	50448637	91.15	*50448637*
Sample 27	RNASeq DCs IL-21	Splenic conventional DC	total RNA	76266388	67221657	88.14	*67221657*
Sample 28	RNASeq DCs IL-10	Splenic conventional DC	total RNA	55779207	50057903	89.74	*50057903*
Sample 29	RNASeq DCs IL-21+LPS	Splenic conventional DC	total RNA	61132659	53315141	87.21	*53315141*
Sample 30	RNASeq DCs IL-10+LPS	Splenic conventional DC	total RNA	71633852	64257370	89.70	*64257370*
Sample 31	RNASeq DCs un-stimulated	Splenic conventional DC	total RNA	55841539	52885686	94.71	*52885686*
Sample 32	RNASeq DCs LPS	Splenic conventional DC	total RNA	61637752	58432964	94.80	*58432964*
Sample 33	RNASeq DCs IL-21	Splenic conventional DC	total RNA	63027015	59112685	93.79	*59112685*
Sample 34	RNASeq DCs IL-10	Splenic conventional DC	total RNA	62783824	59179986	94.26	*59179986*
Sample 35	RNASeq DCs IL-21+LPS	Splenic conventional DC	total RNA	61897924	58252392	94.11	*58252392*
Sample 36	RNASeq DCs IL-10+LPS	Splenic conventional DC	total RNA	51211535	48330117	94.37	*48330117*

Supplementary Table 2. Summary of ChIP-Seq and RNA-Seq libraries
